# Glucan Biosynthesis Protein G Is a Suitable Reference Gene in *Escherichia coli* K-12

**DOI:** 10.5402/2011/469053

**Published:** 2011-11-23

**Authors:** Sean S. J. Heng, Oliver Y. W. Chan, Bryan M. H. Keng, Maurice H. T. Ling

**Affiliations:** ^1^Raffles Institution, One Raffles Institution Lane, Singapore 575954; ^2^Department of Zoology, The University of Melbourne, Genetics Lane, Parkville, Victoria 3010, Australia

## Abstract

The expressions of reference genes used in gene expression studies are assumed to be stable under most circumstances. However, a number of studies had demonstrated that such genes were found to vary under experimental conditions. In addition, genes that are stably expressed in an organ may not be stably expressed in other organs or other organisms, suggesting the need to identify reference genes for each organ and organism. This study aims at identifying stably expressed genes in *Escherichia coli*. Microarray datasets from *E. coli* substrain MG1655 and 1 dataset from W3110 were analysed. Coefficient of variance (COV) of was calculated and 10% of the lowest COV from 4631 genes common in the 3 MG1655 sets were analysed using NormFinder. Glucan biosynthesis protein G (*mdoG*), which is involved in cell wall synthesis, displayed the lowest weighted COV and weighted NormFinder Stability Index for the MG1655 datasets, while also showing to be the most stable in the dataset for substrain W3110, suggesting that *mdoG* is a suitable reference gene for *E. coli* K-12. Gene ontology over-representation analysis on the 39 genes suggested an over-representation of cell division, carbohydrate metabolism, and protein synthesis which supports the short generation time of *E. coli*.

## 1. Introduction

Gene expression analysis is examining the variations in gene expression as a result of changes in environmental conditions by measuring DNA expression levels over time. Quantitative real-time polymerase chain reaction (qRT-PCR) is a commonly used technique to quantify gene expressions [1]. However, several parameters need to be controlled in this process in order to obtain accurate and reliable results. These include variations in the amounts of starting material between samples, RNA extraction efficiency, RNA integrity/quality, efficiency of cDNA synthesis, and differences in the overall transcriptional activity of the cells analyzed. Of which, only the differences in transcriptional activity is of interest. A possible method for accounting other effects is relative normalization, which is the correction of the raw expression values with a reference gene. The reference gene acts as an invariant endogenous control which implies that reference genes should be stably expressed under a wide variety of conditions [2]. 

However, several studies had suggested that it is not easy to find universal reference genes [3–5]. This corroborates several studies demonstrating that several genes originally considered invariable in terms of expression may vary under different experimental conditions [6–8]. For an accurate comparison of DNA expression in different samples, it is necessary to use verified reference genes, such as *GAPDH* (*glyceraldehyde-3-phosphate dehydrogenase*) [9] or *UBQ* (*ubiquinone*) [9], for normalisation or determine new ones for each experimental system with varying external stimuli [3, 10]. However, some studies had also demonstrated that the expression of *GAPDH* [11] and *UBQ* [12] is varying in some conditions. Other studies had also identified references genes, such as *recA *(*recombinase A*), *proC* (*pyrroline-5-carboxylase reductase*) and *gyrA* (*DNA gyrase*) in *Pectobacterium atrosepticum* [8], and *map *(*methionine aminopeptidase*), *rpoC* (*RNA polymerase, beta prime subunit*), and *alaS* (*alanyl-tRNA synthetase*) in *Acidithiobacillus ferrooxidans* [13]. This suggests that established reference genes for a particular organism may not be suitable for other organisms.

*Escherichia coli*, a Gram-negative bacterium commonly found in the gastrointestinal tract, was selected as it has a genome of approximately 4,000 genes. In addition, the genetic material in its plasmids is easily manipulated. Furthermore, *E. coli* is easily cultured and is a commonly studied prokaryotic model [14, 15]. As it is easily cultured in the laboratory environment and is of low pathogenicity [16–18]. 

Candidate reference genes, which are commonly believed to be invariant, can be identified using algorithms such as geNorm [19], NormFinder [20], and BestKeeper [21]. These methods require a wide range of accessible gene expression data, normally obtained through DNA profiling such as quantitative PCR. However, microarrays, which usually contain thousands of probes, present a good source of data for identifying reference genes [22]. A recent study had successfully identified MARK3 as a suitable reference gene in mouse liver using microarray analysis [23].

Currently, there are numerous studies being conducted to validate known reference genes and possibly identify new ones [3, 8, 9, 13, 24]. In this study, we identify and evaluate a set of invariant genes in *E. coli* K-12 substrains MG1655 and W3110. Our results suggest that *glucan biosynthesis protein G* (*mdoG*) is a suitable reference gene for both MG1655 and W3110 strains of *E. coli.*

## 2. Materials and Methods

### 2.1. Microarray Data

Four datasets were obtained from publicly available microarray databases, Gene Expression Omnibus, National Centre for Biotechnology Information, of which 3 were from *E. coli* K-12 substrain MG1655 and 1 from substrain W3110. Briefly, the studies conducted with the datasets are as follows: GDS680: MG1655 grown in either aerobic or anaerobic conditions, deleted for transcriptional regulators in oxygen response, and used to validate a computational model of transcriptional and metabolic networks. GDS1099: aerobically grown MG1655 cells in several media with varied carbon sources including glucose, glycerol, succinate, L-alanine, acetate, and L-proline. GDS1494: analysis of derivatives of strain 1655: wild type, fur mutant, and wild type with added FeSO_4_, induced to overexpress *RyhB*, a noncoding RNA regulated by the fur repressor protein. GDS1827: W3110 cells grown aerobically and exposed to low, neutral, or high pH to study acid and base response.

### 2.2. Finding Invariant Genes

The coefficient of variation (COV) of every gene was calculated as the quotient of standard deviation and arithmetic mean. From 4631 genes, the top 10% with the lowest COV from each dataset were listed. The intersection between the 3 MG1655 data sets (GDS680, GDS1099, and GDS1494) was identified and analysed using NormFinder version 0.953 [20] to rank the stability of these genes. A weighted stability index for each gene was then calculated from the NormFinder's stability index, and an average of the NormFinder stability indexes multiplied by number of samples was taken.

### 2.3. Gene Ontology Overrepresentation Analysis

The list of genes from the intersection of the top 10% with the lowest COV from the 3 MG1655 data sets were analysed for gene ontology overrepresentation using the Gene ontology gene annotation file for *E. coli* dated July 8, 2011. Chi-square test was carried out to identify the overrepresented gene ontology terms in the list of genes using the overall *P* value of 0.01, corrected for multiple testing using Holm-Bonferroni method [25]. 

### 2.4. Comparing NormFinder and COV

Spearman's correlation was used to determine the correlation between stability index generated by NormFinder and COV values using the equation *r* = 1 − [6∑*d*_*i*_^2^/(*n*(*n* ^2^ − 1))], where *r* is the Spearman's correlation, *d* is the difference in the rank of two parameters, and *n* is the sample size. The *t*-statistic was calculated by equation $t=r\sqrt{\left(n-2)/(1-r^{2}\right)}$, which was used to test for the null hypothesis of no correlation with (*n* − 2) degrees of freedom. 

## 3. Results and Discussion

A threshold of less than 10% COV was used to select stably expressed genes across the three datasets GDS 680, 1099, and 1494 (MG1655). A total of 39 genes of consistent low variance were found (Table 1) with the weighted COV values ranging from 0.099 to 0.138. Glucan biosynthesis protein G (*mdoG*) was found to be most stable with both the lowest weighted COV value and weighted NormFinder Stability Index for MG1655. In GDS 1827 (W3110), *mdoG* was the most stable in the dataset, with a COV of 0.088 and NormFinder Stability Index of 0.078. The highest COV in GDS 1827 is 0.791 for *hslV *(*peptidase component of the HslUV protease*). Our results suggest that *mdoG* may be a suitable reference gene across both *E. coli* strains W3110 and MG1655. This may imply that *mdoG *may be suitable for use as reference genes in other strains of *E. coli* K-12. 

Gene ontology overrepresentation is a commonly used mechanistic analysis method to provide biological insights into a list of genes [26–28]. The analysis of the 39 genes with consistently low variance for gene ontology overrepresentation showed that 3 primary functions were found to be overrepresented (Table 2). They were cell division, carbohydrate metabolic process, and protein synthesis. As *E. coli* is generally accepted as a rapidly dividing prokaryote [29], it is plausible to expect genes responsible for cell division to be constantly expressed. As the cells grow, new cellular structures, such as cell wall and other enzymes, need to be synthesized. Hence, it is plausible to expect protein synthesis to be stable throughout the cell cycle. The role of glutathione [30, 31] and tetrapyrrole [32] had been implicated in protein synthesis while diaminopimelate had been shown to have a role in the maintenance of cell wall [33]. At the same time, cell division involves the replication and segregation of genetic material [34]. Carbohydrate is both a primary source of energy for *E. coli* [35] as well as the primary component of bacterial cell wall [36]. The gene *mdoG *has been shown to be involved in the formation of the *β*-1,6 glucose linkage [37] and in the periplasmic release of newly synthesized osmoregulated periplasmic glucans [38, 39], which is needed for bacterial cell wall. Thus, it is plausible that the expression of *mdoG* is needed during binary fission. As *E. coli *divides rapidly, constant synthesis of cell wall is needed. Therefore, it is likely that *mdoG* is constantly needed, which may be a reason to its constant expression in *E. coli. *Hence, both gene ontology overrepresentation and the function of the most stably expressed gene, *mdoG*, support the short generation time of *E. coli*. 

Our results showed that none of the 7 housekeeping genes consistently appeared in the lowest 10% COV subset of each dataset (Table 3), while *GAPDH *[9], *gyrA *[8], and *alaS* [13] were found to be in the lowest 10% COV subset, in one dataset each. Our results illustrated that *recA *[8] has the highest weighted COV of 0.5378 and *gyrA *[8] has the lowest weighted COV of 0.1607, which is higher than that of *mdoG* (COV of 0.099). This suggests that commonly used housekeeping genes such as *GAPDH *[9] and *recA* [8] are not suitable for the expression profiling of *E. coli*. Hence, our results support our earlier hypothesis that common housekeeping genes found to be stable in one organism cannot be assumed to be stable in all organisms. This suggests the need to identify suitable reference genes for each organism of interest.

The advantage of COV is its capability to analyse as large number of samples as required [23] as the number of calculations increases proportionally to the sample size, resulting in linear complexity. NormFinder uses residual analysis between sample subgroup variation and the overall variation of the expression dataset to evaluate the variation contributed by each gene in the entire dataset [20]. Thus, the computational complexity of NormFinder increases exponentially as the number of samples increases; hence, it is only able to work with a small number of genes within reasonable time and computational resources. Therefore, we used Spearman's rank correlation coefficient to determine the correlation of stability index by NormFinder and COV values which showed that the sum of *d* ^2^ was 5664 and the *P* value was 0.006748. Since the *P* value was less than 0.01, the null hypothesis is rejected, indicating that there is correlation between the stability index from NormFinder and COV values but the strength of this correlation is difficult to establish as the significance in *P* value did not indicate the correlation strength. However, our results do not suggest that COV is a suitable replacement for NormFinder. As NormFinder [20] takes account of the overall variability in the entire dataset, it is likely to be statistically stronger than COV which is a normalized standard deviation. Given the advantageous ability of COV to process large amounts of data such as those derived from microarrays, it is plausible that COV can be used as a weaker filter for a broad category of genes with low expression variation, followed by stronger statistical analysis by NormFinder [20] to identify suitable reference genes.

## Figures and Tables

**Table 1:** Weighted mean COV and NormFinder stability index of 39 invariant genes across 3 datasets (MG1655).

Gene symbol	Gene name	Weighted COV values	Weighted NormFinder Stability Index
*mdoG*	Glucan biosynthesis protein G	0.099	0.082
*dapA*	Dihydrodipicolinate synthase	0.106	0.090
*crp*	DNA-binding transcriptional dual regulator	0.106	0.102
*hslV*	Peptidase component of the* HslUV *protease	0.111	0.105
*mrdB*	Cell wall shape-determining protein	0.101	0.114
*fucU*	L-Fucose mutarotase	0.107	0.117
*yjgP*	LPS transport* (lptF) *	0.105	0.117
*yigC*	3-Octaprenyl-4-hydroxybenzoate decarboxylase	0.126	0.117
*sun*	16S rRNA m(5)C967 methyltransferase, S-adenosyl-L-methionine-dependent	0.130	0.119
*gor*	Glutathione oxidoreductase	0.117	0.126
*hflB*	ATP-dependent metalloprotease	0.127	0.130
*yqiB*	Predicted dehydrogenase	0.125	0.134
*murG*	N-Acetylglucosaminyl transferase	0.124	0.134
*yrbG*	Predicted calcium/sodium:proton antiporter	0.122	0.134
*yejK*	Nucleotide associated protein	0.120	0.141
*yfgA*	Cytoskeletal protein required for* MreB *assembly	0.118	0.142
*hflX*	Putative GTPase* HflX *	0.105	0.142
*spoT*	Bifunctional (*p*)ppGpp synthetase II/guanosine- 3′,5′-bis pyrophosphate 3′-pyrophosphohydrolase	0.117	0.143
*holC*	DNA polymerase III, chi subunit	0.134	0.144
*xerD*	Site-specific tyrosine recombinase	0.114	0.146
*tolB*	Periplasmic protein	0.115	0.146
*yheS*	Fused predicted transporter subunits of ABC superfamily: ATP-binding components	0.110	0.146
*ntpA*	Dihydroneopterin triphosphate pyrophosphatase	0.118	0.147
*yabB*	Conserved protein, *MraZ *family	0.115	0.148
*lolA*	Chaperone for lipoproteins	0.117	0.153
*yggD*	Predicted DNA-binding transcriptional regulator	0.116	0.153
*pnp*	Polynucleotide phosphorylase/polyadenylase	0.110	0.155
*yrbB*	ABC transporter maintaining OM lipid asymmetry, cytoplasmic STAS component	0.123	0.156
*rnc*	RNase III	0.117	0.157
*xerC*	Site-specific tyrosine recombinase	0.138	0.160
*rfaF*	ADP-heptose:LPS heptosyltransferase II	0.120	0.161
*yigP*	Conserved protein, SCP2 family	0.122	0.164
*gyrB*	DNA gyrase, subunit B	0.126	0.164
*nagC*	DNA-binding transcriptional dual regulator, repressor of N-acetylglucosamine	0.132	0.165
*nrdR*	Conserved protein	0.118	0.168
*hemD*	Uroporphyrinogen III synthase	0.108	0.169
*pheT*	Phenylalanine tRNA synthetase, beta subunit	0.124	0.171
*frr*	Ribosome recycling factor	0.129	0.173
*cls*	Cardiolipin synthase 1	0.129	0.181

**Table 2:** Gene ontology overrepresentation of the 39 invariant genes.

Primary function	GOID	Gene ontology terms	*P* value
Cell division	GO:0071139	Cell cycle	5.51*E* − 61
	GO:0006276	Plasmid recombination	6.01*E* − 31
	GO:0016051	Cell division	2.90*E* − 16
	GO:0006432	Plasmid maintenance	2.90*E* − 16
	GO:0042594	Response to starvation	3.39*E* − 11
	GO:0007049	Chromosome segregation	6.61*E* − 09
	GO:0017038	Resolution of recombinant intermediates	1.21*E* − 08
	GO:0006004	Guanosine tetraphosphate metabolic process	1.21*E* − 08
	GO:0042953	Lysogeny	4.20*E* − 07
	GO:0051301	Diaminopimelate biosynthetic process	3.50*E* − 06

Carbohydrate metabolism	GO:0030259	Carbohydrate biosynthetic process	2.34*E* − 31
	GO:0006749	Lipid glycosylation	2.34*E* − 31
	GO:0007059	Fucose metabolic process	8.08*E* − 13

Protein synthesis	GO:0030069	Phenylalanyl-tRNA aminoacylation	2.34*E* − 31
	GO:0016075	RNA catabolic process	2.34*E* − 31
	GO:0015969	Lipoprotein transport	2.90*E* − 16
	GO:0019277	Phenylalanyl-tRNA aminoacylation	2.90*E* − 16
	GO:0042150	Protein import	3.39*E* − 11
	GO:0006396	RNA processing	2.48*E* − 09
	GO:0033014	Tetrapyrrole biosynthetic process	1.21*E* − 08
	GO:0019877	Glutathione metabolic process	4.20*E* − 07

**Table 3:** Seven housekeeping genes and their mean COV values across 4 datasets.

Gene symbol	Gene name	Weighted COV values
*recA*	*Recombinase A*	0.537752244
*proC*	*Pyrroline-5-carboxylate reductase*	0.257211257
*gyrA*	*DNA gyrase*	0.16070208
*map*	*Methionine aminopeptidase*	0.282917613
*rpoC*	*RNA polymerase, beta prime subunit*	0.273422333
*alaS*	*Alanyl-tRNA synthetase*	0.160829965
*GAPDH*	*Glyceraldehyde-3-phosphate dehydrogenase*	0.230513521
